# Chocolate Processed by Extrusion Cooking Affects Glucose and Lipid Metabolism in Humans: A Randomized, Double‐Blind, Crossover Trial

**DOI:** 10.1002/fsn3.72409

**Published:** 2026-09-30

**Authors:** Yasuyoshi Kinta, Hiroko Maruki‐Uchida, Masahiro Umehara, Ryoichi Ito, Ryotaro Shiga, Yuki Yasumoto, Sadao Mori, Takashi Koikeda

**Affiliations:** ^1^ Research Institute Morinaga & Co. Ltd. Yokohama Kanagawa Japan; ^2^ Shiba Palace Clinic Tokyo Japan

**Keywords:** blood sugar, chocolate, extrusion, food processing, metabolism, triglycerides

## Abstract

Promoting positive and well‐being‐centered eating behavior in humans has recently garnered considerable attention. If the postprandial metabolic response of nutrients, especially sugars that are deleterious to health, can be controlled through processing, we can enjoy food without restrictions. To our knowledge, no study has reported the effect of extrusion cooking on the digestive response of sugars other than starch. Hence, we aimed to examine the effects of consuming extrusion‐processed chocolate on glucose, insulin, and triglyceride blood levels through a randomized, double‐blind, crossover trial using compositionally identical chocolate processed without extrusion. We hypothesized that extrusion processing is associated with the postprandial metabolic response of sugars. Participants who consumed chocolate processed by extrusion cooking exhibited typically lower blood glucose concentration (17.8 ± 6.0 mg/dL) at 45 min and significantly lower blood insulin (20.6 ± 3.1 μU/mL) at 30 min than those who consumed chocolate processed without extrusion (25.1 ± 6.4 mg/dL and 26.8 ± 3.9 μU/mL, respectively). Serum triglyceride concentrations continued to increase until 180 min following chocolate ingestion, with levels in participants who consumed extrusion‐processed chocolate being significantly lower than those in the group that consumed chocolate processed without extrusion. The appetite survey revealed a sustained feeling of fullness after consuming extrusion‐processed chocolate. In simulated dissolution, extrusion‐processed chocolate yielded less sucrose equivalent than processing‐free chocolate, indicating a dissolution rate influence on human digestion. In conclusion, our findings suggest that glucose and lipid metabolism can be controlled through food processing, which has implications for the development of simultaneously palatable and healthy foods.

## Graphical abstract

The benefits of extrusion‐processed chocolate helped establish a correlation between food processing and glucose and lipid metabolism, enabling the development of palatable and healthy foods to promote overall well‐being.

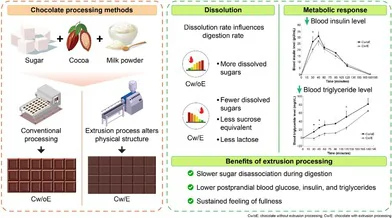

## 1 Introduction

Although sugars and lipids are important nutritional components, they can cause various health problems depending on how they are consumed. If energy intake continues to exceed energy expenditure, excess energy may accumulate as fat, leading to obesity and metabolic syndrome, both of which are linked to diabetes and cardiovascular disease (Bovolini et al. 2021; Hamooya et al. 2025; Kim et al. 2021; Kovacs and Hajnal 2022; Mishra et al. 2021; Stefan and Schulze 2023). Hyperglycemia, which is closely related to sugar intake, negatively impacts health and increases the risk of cardiovascular disease mortality (Hyvärinen et al. 2009; Kanter et al. 2021). Impaired glucose tolerance is a more influential risk factor for hyperglycemia than impaired fasting glucose (Okamura et al. 2024). Moreover, fluctuating blood glucose levels, which describe rapid increases in blood glucose levels within a short period after a meal, are deleterious to endothelial function and oxidative stress, leading to cardiovascular disease (Ahn et al. 2023; Ceriello et al. 2008; Huang et al. 2023; Shuto et al. 2015). Thus, optimum control of blood glucose levels is especially important.

A more positive and well‐being‐centered approach to human eating behavior has been proposed, moving beyond considering food as pure nourishment (Block et al. 2011; Cornil and Chandon 2016; Renner et al. 2012; Wahl et al. 2017). This perspective promotes a holistic view of food in overall well‐being, moving away from restraint and health restriction (Block et al. 2011). It suggests that health solutions should consider not only nutrient quantity but also how intake methods and food types affect nutrient utilization.

Food processing by extrusion, which involves continuous mixing and kneading, is widely used to produce snacks and cereals owing to its versatility, low cost, rapid processing, and high‐temperature and high‐pressure compatibility (Brennan et al. 2011). Extrusion processing controls the digestive response of nutritional components. For example, studies using starch reported improved digestibility following extrusion processing owing to increased gelatinization (Wang et al. 2023; Ye et al. 2018). Robin et al. (2016) studied the digestibility of different maize starches and reported that an increase in amylose content was correlated with lower slowly digestible starch content in native starches; however, this trend was reversed after extrusion. Wang et al. (1993) used lipids and reported that total fat content was not significantly altered in extruded whole wheat; however, only 50% of ether‐extractable lipids were detected, suggesting that the starch–lipid complexes formed during extrusion are resistant to some lipid extraction procedures. Ito et al. (2016) reported that the amount of fecal triacylglycerol excreted in rats fed cornstarch extruded with soybean oil was higher than that in rats fed cornstarch extruded without soybean oil but with the same diet composition, which was attributed to low solvent extraction rates. Thus, the digestive response of nutritional components can be controlled by extrusion processing. Although previous studies have reported the process‐related degradation of sugar components (Moreno et al. 2018), to our knowledge, no studies have reported the effect of extrusion on the digestive response of sugar, which is deleterious to health. For sugars, extrusion processing may also control the dissolution/release, which is attributed to extraction rates, as reported for lipids by Wang et al. (1993) and Ito et al. (2016).

In this study, we hypothesized that extrusion processing would be associated with postprandial metabolic response and a resistance to some water dissolution procedures of sugars. To test this, we analyzed the effects of extrusion cooking on the postprandial metabolic response of the nutritional components in chocolate, and on resistance to dissolution. Chocolate is a typical sugar‐containing sweet whose palatability is also influenced by psychological factors (Bae et al. 2024; Nasser et al. 2011; Orsolini et al. 2022). In addition to placing less emphasis on restricting intake, the concept of food well‐being includes the psychological effects of consumption. In this study, we aimed to investigate the effects of consuming extrusion‐processed chocolate on blood levels of glucose, insulin, and triglycerides in humans by comparing two compositionally similar chocolates, partially processed with and without extrusion, along with surveying the feelings of appetite. Furthermore, we aimed to investigate the effects of extrusion processing on resistance to some water dissolution procedures and to explore the potential underlying mechanisms.

## 2 Materials and Methods

### 2.1 Clinical Evaluation of the Effects of Chocolate Consumption on Blood Glucose, Insulin, and Triglyceride Levels

#### 2.1.1 Protocol

This randomized, double‐blinded trial was conducted in 18 healthy Japanese men aged between 20 and 60 years. The study was approved by the ethics committee of the Shiba Palace Clinic (157022–38759) and conducted in accordance with the ethical principles of the Declaration of Helsinki (revised in 2013) and the Ethical Guidelines for Life Sciences and Medical Research Involving Human Subjects (Ministry of Education, Culture, Sports, Science, and Technology; Ministry of Health, Labor, and Welfare; and Ministry of Economy, Trade, and Industry, Japan) in January 2025. Written informed consent was obtained from all participants after a thorough explanation of the study purpose, content, and procedures. The study followed the Consolidated Standards of Reporting Trials (CONSORT) guidelines (Figure 1). This study was registered in the University Hospital Medical Information Network Clinical Trial Registry (Japan, registration no. UMIN000056919) on February 3, 2025, and disclosed on July 1, 2025. Details are available at the following URL: https://center6.umin.ac.jp/cgi‐open‐bin/ctr_e/ctr_view.cgi?recptno = R000064980. The study was performed according to the registered protocol on two different days separated by a washout period of more than 1 week in February 2025.

**FIGURE 1:**
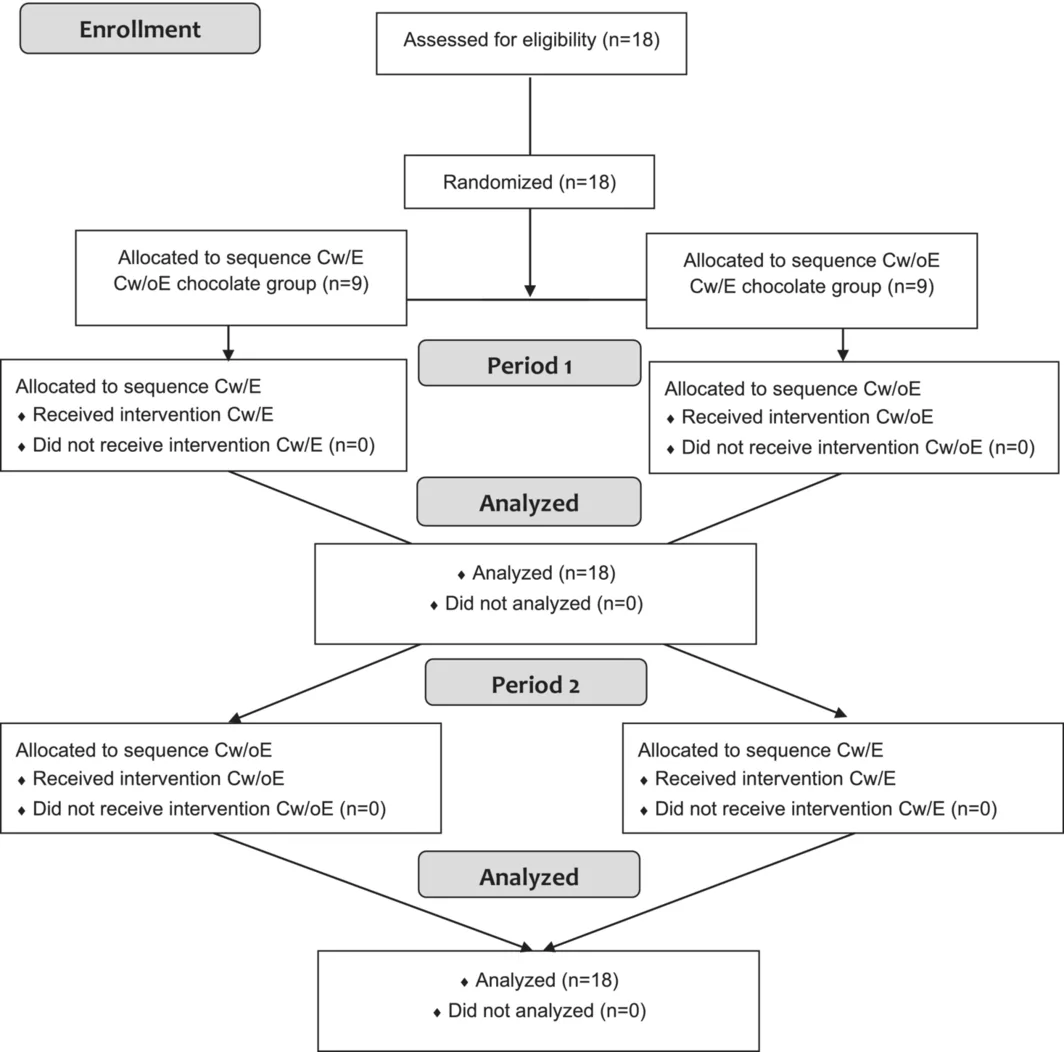
CONSORT flow diagram of the study design.

#### 2.1.2 Participants

The inclusion criteria were as follows: (1) Japanese men aged between 20 and 60 years at the time of obtaining consent; (2) participants who were fit for clinical visits on the designated date and able to provide multiple blood samples (7 samples/day × 2 observations); (3) healthy participants (with no serious organ damage or disease and not receiving any treatment related to them or any type of drug therapy based on the participant's report at the start of the study); (4) participants who were fully informed of the purpose and content of the study, capable of understanding the study, able to voluntarily participate in the study, and able to provide written consent to participate in the study; and (5) participants with a body mass index between 20.0 and 24.9.

The exclusion criteria for participants were as follows: (1) those with chronic diseases or disorders of the liver, biliary tract, digestive system, circulatory system, respiratory system, kidney, urinary system, brain, or nervous system; (2) those allergic to medicines or food; (3) habitual smokers; (4) those vulnerable to lifestyle changes during the study period (e.g., work at night, travel for long periods); (5) those visiting the hospital, taking medication, or undergoing medical treatment; (6) those with lifestyle‐related diseases (such as diabetes, hypertension, and dyslipidemia), including those with fasting blood glucose levels in the diabetic range (≥ 126 mg/dL) or those with a past disease diagnosis; (7) those with severe anemia; (8) those who dislike chocolate; (9) those taking drugs for disease treatment in the past month (excluding those taking medication for headache, common cold, etc.); (10) those receiving treatment involving hospitalization within the past 6 months; (11) habitual heavy drinkers (more than 60 g of alcohol per day e.g., 1500 mL of beer, 2.5 g of sake, and approximately 200 mL of whiskey straight); and (12) those currently participating in another clinical trial, intending to participate in another clinical trial within 4 weeks after completion of the current trial, or intending to participate in another clinical trial after agreeing to participate in the current trial. The clinical characteristics of the participants are presented in Table 1.

**TABLE 1:** Baseline characteristics of the study participants (*n* = 18).

Characteristic	Mean ± standard deviation
Age, years	48.7 ± 7.2
Height, cm	169.5 ± 7.7
Body weight, kg	65.0 ± 8.6
Body mass index, kg/m^2^	22.5 ± 1.6

#### 2.1.3 Test Samples

Two chocolate samples were prepared for the experiments: chocolate with extrusion processing (Cw/E) and chocolate without extrusion processing (Cw/oE). For Cw/E, part of the raw material was extrusion‐processed in advance before preparing the chocolate. The extrusion processing and chocolate formulations are presented in Table 2.

**TABLE 2:** Extrusion processing and formulation of chocolate with extrusion processing (Cw/E) and chocolate without extrusion processing (Cw/oE) samples.

Process	Ingredient	Cw/E	Cw/oE
With extrusion	Sugar	33.55	
	Cocoa mass	6.60	
	Whole milk powder	14.85	
Without extrusion	Sugar		33.55
	Cocoa mass		6.60
	Whole milk powder	9.90	24.75
	Skim milk powder	11.00	11.00
	Cocoa butter equivalent	24.00	24.00
	Lecithin	0.10	0.10
	Total	100.00	100.00

Extrusion experiments were performed using a laboratory‐scale twin screw co‐rotating extruder (ZSK34Mv Plus, Coperion, Germany) with a barrel diameter of 35 mm, a length‐to‐diameter ratio of 44, and a die diameter of 13 mm. The extruder was evenly divided into 11 zones, and the temperature for each zone except one was set independently as follows: NA, 40°C, 60°C, 95°C, 95°C, 40°C, 40°C, 20°C, 20°C, 20°C, and 20°C. Extruder screw speed for the experimental treatments was set to 200 rpm. Among the raw materials, powdered materials were fed into the extruder from zone one, whereas pre‐melted cocoa mass and water were fed from zone two. The feed rates of sugar plus whole milk powder, cocoa mass, and water were 26.2, 3.58, and 0.61 kg/h, respectively. The moisture was adjusted to 0.05 MPa in zone seven. The extruder terminated in a front die plate wherein the entire extruded mixture was passed through a 13 mm‐diameter orifice at approximately 105°C, which was then cut at 1400 rpm. Extrudates were ground through the 1 mm screen of the pin mill and subjected to further chocolate preparation.

Chocolate was prepared by refining, using a roll refiner, and conching from sugar (33.55%), cocoa mass (6.6%), whole milk powder (24.75%), skim milk powder (11%), cocoa butter equivalent (24%), and lecithin (0.1%) according to conventional methods (Beckett 2009; Minifie 1989). A solid chocolate sample was prepared by tempering using a marble slab. The tempering procedure was as follows: a bowl of chocolate was melted at 40°C, and two‐thirds of this chocolate was turned over on a marble slab with a scraper until the chocolate cooled to approximately 28°C. The chocolate was added back into the bowl and mixed thoroughly using a silicone spatula. The final temperature of the well‐tempered chocolate was approximately 30°C. For solidification, the tempered chocolates were poured into polycarbonate molds and cooled to 10°C for 30 min. The samples were removed from the molds after cooling; the resulting cuboid‐like chocolate samples (weighing 3.8 g each) were subjected to further analyses. All samples were prepared in a sanitary environment in the laboratory of Morinaga & Co. Ltd. The nutrient analysis results of the test samples are presented in Table 3.

**TABLE 3:** Nutrient analysis of the test samples (per 100 g).

Component	Cw/E	Cw/oE	Analysis method
Protein (g)	11.1	11.3	Combustion
Total fat (g)	33.6	34.1	Acid hydrolysis
Cholesterol (mg)	25	26	GC
Ash (g)	2.5	2.3	Direct ashing
Water (g)	0.8	0.7	Vacuum oven drying
Fructose (g)	Not detected	Not detected	HPLC (limit of quantitation = 0.5)
Galactose (g)	Not detected	Not detected	
Glucose (g)	Not detected	Not detected	
Lactose (g)	14.9	14.5	
Maltose (g)	Not detected	Not detected	
Sucrose (g)	33.5	33.5	
Starch (g)	0.6	0.5	Enzyme
Dietary fiber (g)	0.9	0.8	Enzymatic–gravimetric
Sodium (mg)	120	120	ICP atomic emission spectroscopy

Abbreviations: Cw/E, chocolate with extrusion processing; Cw/oE, chocolate without extrusion processing; GC, gas chromatography; HPLC, high‐performance liquid chromatography; ICP, inductively coupled plasma.

#### 2.1.4 Consumption of Chocolate Samples and Postprandial Blood Analysis

After fasting for 12 h, blood samples of all participants were collected before and after ingestion (0, 30, 45, 60, 90, 120, and 180 min). The time points for blood collection were determined based on previous studies of blood glucose and triglyceride levels (Jameel et al. 2014; Petyaev et al. 2019; Shimomura et al. 2005). Each participant consumed 20 pieces of chocolate (76 g) within 5 min without water. Next, they consumed 100 mL of water, and after 20 min of ingestion, participants were allowed to consume water freely. The blood samples were centrifuged and serum was obtained for further analysis. The serum samples were stored at 4°C. All blood data were analyzed at the Medience Corporation (Tokyo, Japan). Blood glucose and triglyceride levels were measured enzymatically using glucose oxidase and via free glycerol scavenging. Insulin levels were measured using chemiluminescence immunoassay.

#### 2.1.5 Questionnaires

The appetite questionnaire included questions on the visual analog scale (in mm) developed for this study regarding the participant's feelings of hunger, fullness, and desire to eat. The questionnaire was assessed before (0 min) and after (60, 120, and 180 min) ingestion and blood collection. Opposing extremes for each feeling were described at either end of a 100 mm horizontal line, with participants marking the line to indicate how they felt at that moment.

### 2.2 In Vitro Evaluation of the Effects of Salivary and Gastric Fluids on Chocolate Composition

#### 2.2.1 Chemicals

Potassium chloride, potassium dihydrogen phosphate, sodium hydrogen carbonate, sodium chloride, magnesium chloride hexahydrate, ammonium carbonate, calcium chloride dihydrate, sucrose, lactose monohydrate, 1 mol/L hydrochloric acid, and high‐performance liquid chromatography (HPLC)‐grade acetonitrile were obtained from Wako Chemicals (Osaka, Japan). Fructose and glucose were obtained from Nacalai Tesque (Kyoto, Japan).

#### 2.2.2 Composition of Simulated Dissolving Solution in the Stomach

The compositions of simulated dissolving solution of saliva and gastric juice are presented in Table 4. The simulated fluids were adopted from Brodkorb et al. (2019) with slight modifications. Digestive enzymes (amylase and pepsin) were not added because the focus of the experiment was monosaccharide and disaccharide dissolution.

**TABLE 4:** Composition of simulated dissolving solution of saliva (SDSS) and simulated dissolving solution of gastric juice (SDSG).

Constituent	SDSS [g/L]	SDSG [g/L]
KCl	1.126	1.028
KH_2_PO_4_	0.503	0.244
NaHCO_3_	1.142	4.200
NaCl	—	5.520
MgCl_2_(H_2_O)_6_	0.030	0.040
(NH_4_)_2_CO_3_	0.006	0.148

#### 2.2.3 Sample Preparation

Sample preparation was performed according to the method of Wang et al. (2021). Simulated dissolving solution in the stomach (3.75 mL; comprising 291.2 g/L each of simulated dissolving solution of saliva and gastric juice, 0.077 g/L of CaCl_2_(H_2_O)_2_, and 258.5 g/L of 1 mol/L HCl) was added to melted chocolate (1.25 g) and incubated at 37°C for 5, 15, and 30 min. After incubation, the supernatant liquid (1.4 mL) was centrifuged (20°C, 1 min, 15,000 × ***g***). The obtained supernatant liquid was diluted 1.5 times with 0.1 M phosphate buffer (pH 7.4) and centrifuged (20°C, 10 min, 15,000 × ***g***). Next, the obtained supernatant liquid was diluted 1.5 times with pure water and centrifuged (20°C, 10 min, 15,000 × ***g***). Finally, the obtained supernatant liquid was analyzed using HPLC. The amount of HCl (1 mol/L) was set after all the chocolate had dissolved to obtain a pH value of approximately 3.

#### 2.2.4 Quantitative HPLC Analysis of Fructose, Glucose, Sucrose, and Lactose in Simulated Dissolving Solution in the Stomach

Sucrose and lactose release from the chocolate into the simulated dissolving solution in the stomach was analyzed using HPLC according to Unno (2015). The sucrose equivalent, defined as the total molarity of sucrose and glucose, was used to quantify sucrose because the sucrose was hydrolyzed into glucose and fructose during incubation. The analysis was performed using a Unison UK‐amino column (250 × 4.6 mm i.d., particle size: 3 μm, Imtakt, Kyoto, Japan) and an HPLC system (autosampler: AS‐2057, pump: PU‐2080, column oven: CO‐2065, RI detector: RI‐4030, JASCO Corporation, Tokyo, Japan). The mobile phase was acetonitrile: water (80:20 v/v). The column oven temperature and flow rate were set to 60°C and 0.6 mL/min, respectively. The data were analyzed using ChromNAV (version 1.18.04, JASCO Corporation). Calibration curves of the standard fructose, glucose, sucrose, and lactose were prepared using known concentrations (133.5, 139.4, 139.6, and 132.2 mM) and established using an RI detector. The calibration curves showed correlation coefficients of > 0.997, 0.997, 0.997, 0.996 in the millimole ranges of 0.13–16.69, 0.07–17.43, 0.07–17.46, and 0.06–16.53 mM.

### 2.3 Statistical Analyses

Data are expressed as the mean ± standard error of the mean. All statistical analyses were performed using the free statistical software, EZR (Version 1.65) (Kanda 2013). The level of statistical significance was set to *p* < 0.05.

#### 2.3.1 Clinical Study

The differences from baseline values were compared using the Wilcoxon signed‐rank test. The incremental areas under the curves (iAUC) for glucose, insulin, and triglycerides were geometrically calculated using the trapezoidal rule and compared among the intervention treatments using the paired‐*t*‐test. Recruitment of more than 10 participants is recommended to calculate the food GI (Granfeldt et al. 2006; Nantel 1999). The sample size of this study was determined using EZR by considering previous studies and assuming a significance level of 0.05 and 80% power (Dall'Asta et al. 2022; Shimomura et al. 2005; Tuccinardi et al. 2022). Accounting for withdrawals, the total sample size was determined as 18.

#### 2.3.2 In Vitro Study

The concentrations of sucrose equivalent and lactose in the simulated dissolving solution in the stomach were compared at each time point during incubation using Welch's test.

## 3 Results

### 3.1 Clinical Evaluation of the Effects of Chocolate Consumption on Blood Glucose, Insulin, and Triglyceride Levels

The trial was completed in 18 healthy male participants. Basal fasting blood glucose, insulin, and triglyceride levels were 95.8 ± 2.1 mg/dL, 7.0 ± 0.8 μU/mL, and 95.1 ± 13.3 mg/dL in Cw/oE, respectively, and 95.6 ± 2.0 mg/dL, 6.3 ± 0.6 μU/mL, and 111.3 ± 14.6 mg/dL in Cw/E. These levels were not significantly different between treatments (*p* = 0.947, 0.299, and 0.123, respectively). Figure 2 shows the concentration profiles for plasma glucose, serum insulin, and serum triglyceride levels after ingestion of Cw/E or Cw/oE. Blood glucose levels in the Cw/E group decreased from 19.6 ± 4.3 mg/dL (30 min) to 17.8 ± 6.0 mg/dL (45 min), whereas those in the Cw/oE group increased from 23.6 ± 4.4 mg/dL (30 min) to 25.1 ± 6.4 mg/dL (45 min). At 45 min, the blood glucose concentration of the Cw/E group was typically lower than that of the Cw/oE group. Blood insulin levels in the Cw/E group increased from 20.6 ± 3.1 μU/mL (30 min) to 27.4 ± 3.7 μU/mL (45 min), whereas those in the Cw/oE group increased from 26.8 ± 3.9 μU/mL (30 min) to 31.3 ± 3.8 μU/mL (45 min). At 30 min, the blood insulin concentration in the Cw/E group was significantly lower than that in the Cw/oE group (*p* = 0.033). Serum triglyceride concentrations continued to increase until 180 min following chocolate ingestion, with levels in the Cw/E group being significantly lower than those in the Cw/oE group at 30 and 45 min (*p* = 0.048 and 0.044, respectively).

**FIGURE 2:**
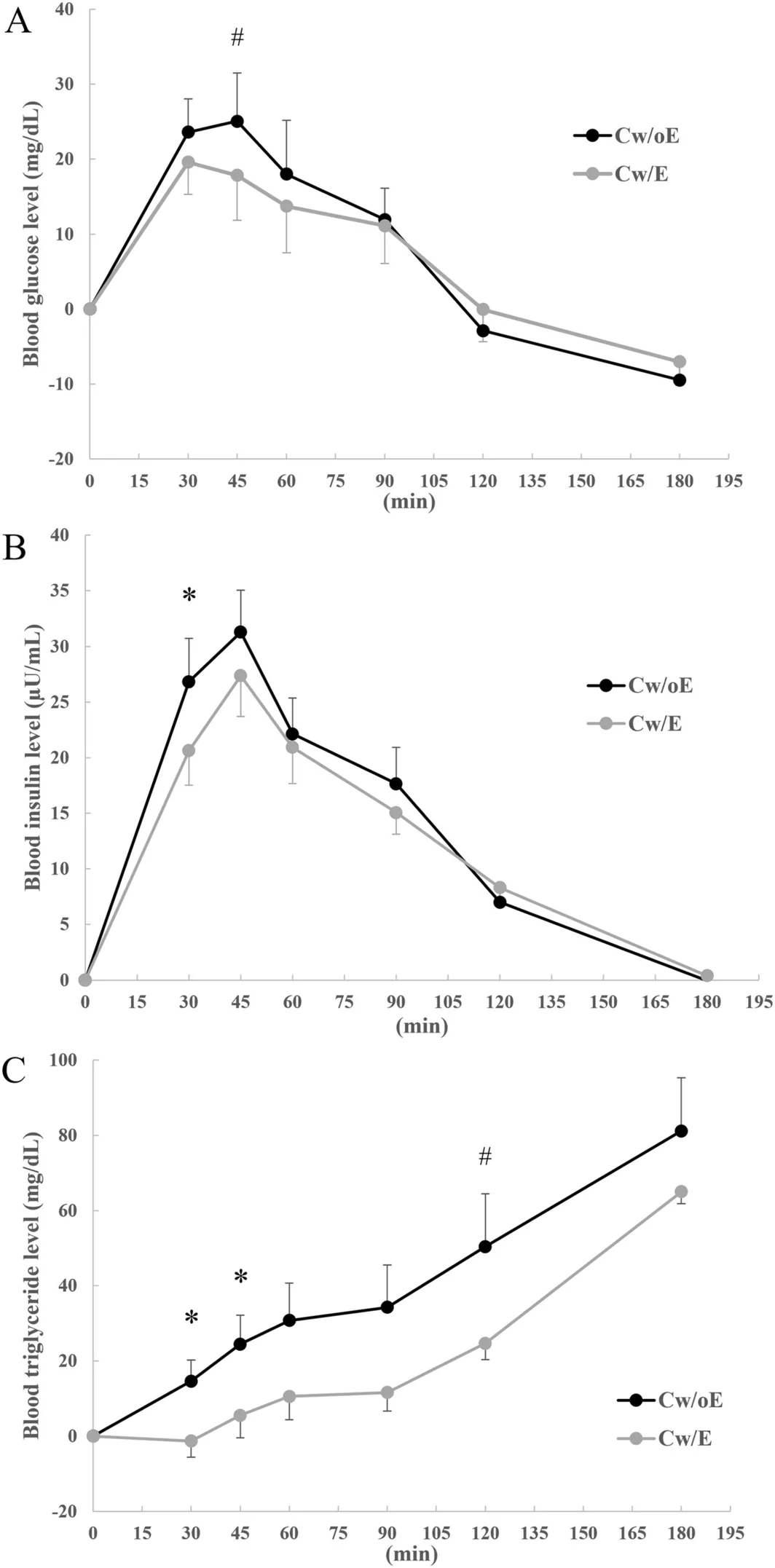
Time‐dependent changes in (A) plasma glucose, (B) serum insulin, and (C) serum triglyceride levels following the consumption of chocolate processed with or without extrusion (Cw/E or Cw/oE, respectively). Error bars indicate the standard error of the mean (SEM). *p < 0.05; #*p* < 0.1, difference between two groups.

The iAUC values for glucose, insulin, and triglycerides were lower in the Cw/E group than in the Cw/oE group (Figure 3); however, only the iAUC values for insulin showed a significant difference between treatments at 30, 45, 60, 90, and 120 min (*p* = 0.025, 0.009, 0.006, 0.016, and 0.040, respectively).

**FIGURE 3:**
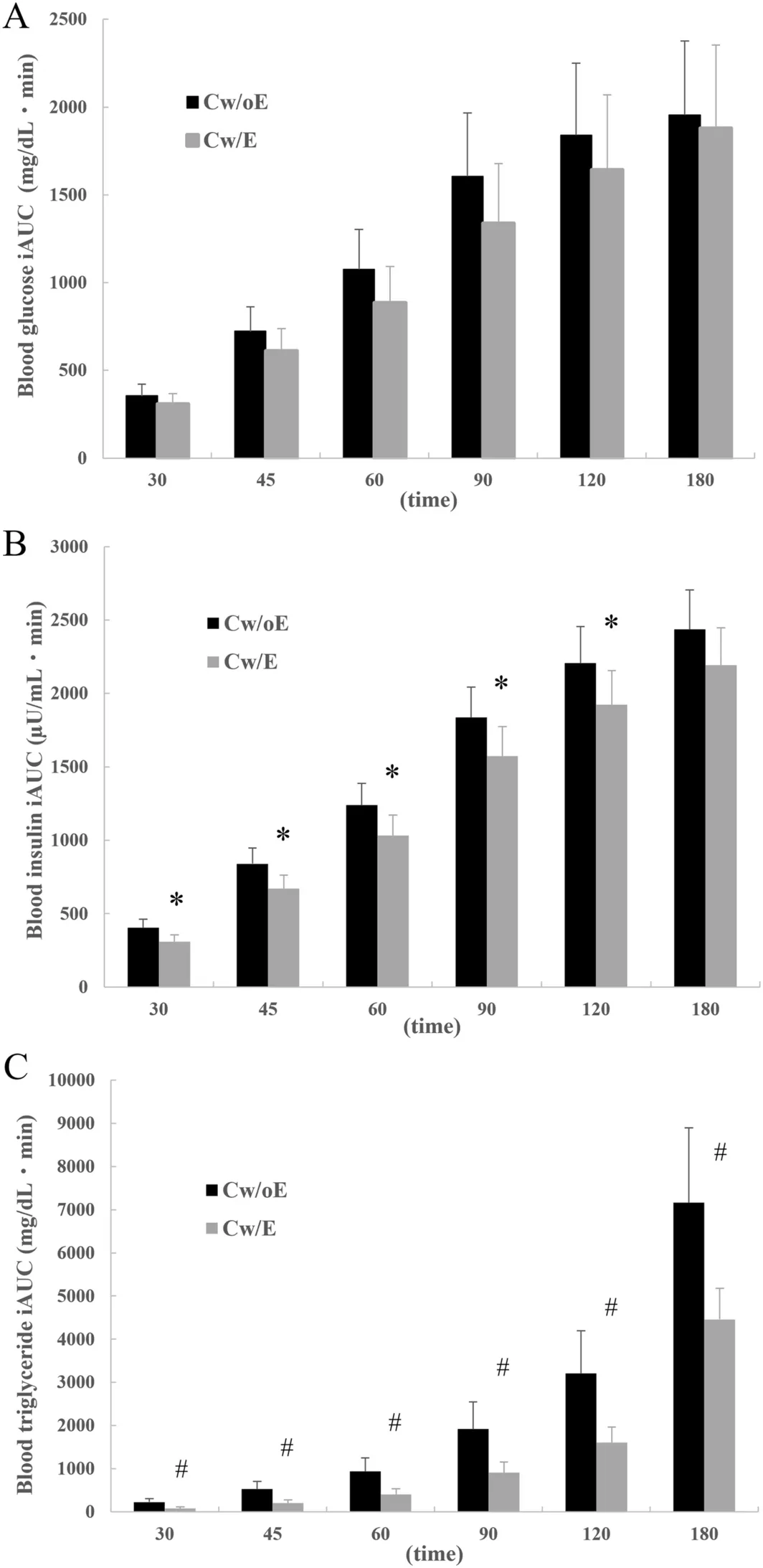
Time‐dependent incremental areas under the curves (iAUC) for changes in the levels of (A) plasma glucose, (B) serum insulin, and (C) serum triglyceride levels following the consumption of chocolate processed with or without extrusion (Cw/E or Cw/oE, respectively). Error bars indicate SEM. *p < 0.05; #*p* < 0.1, difference between two groups.

Based on the questionnaires, chocolate consumption decreased feelings of hunger and desire to eat, rather than increasing feelings of fullness. In terms of participant responses, no significant differences were observed between the Cw/E and Cw/oE groups, except for feelings of fullness at 120 min, which were higher in the Cw/E group (*p* = 0.035; Figure 4).

**FIGURE 4:**
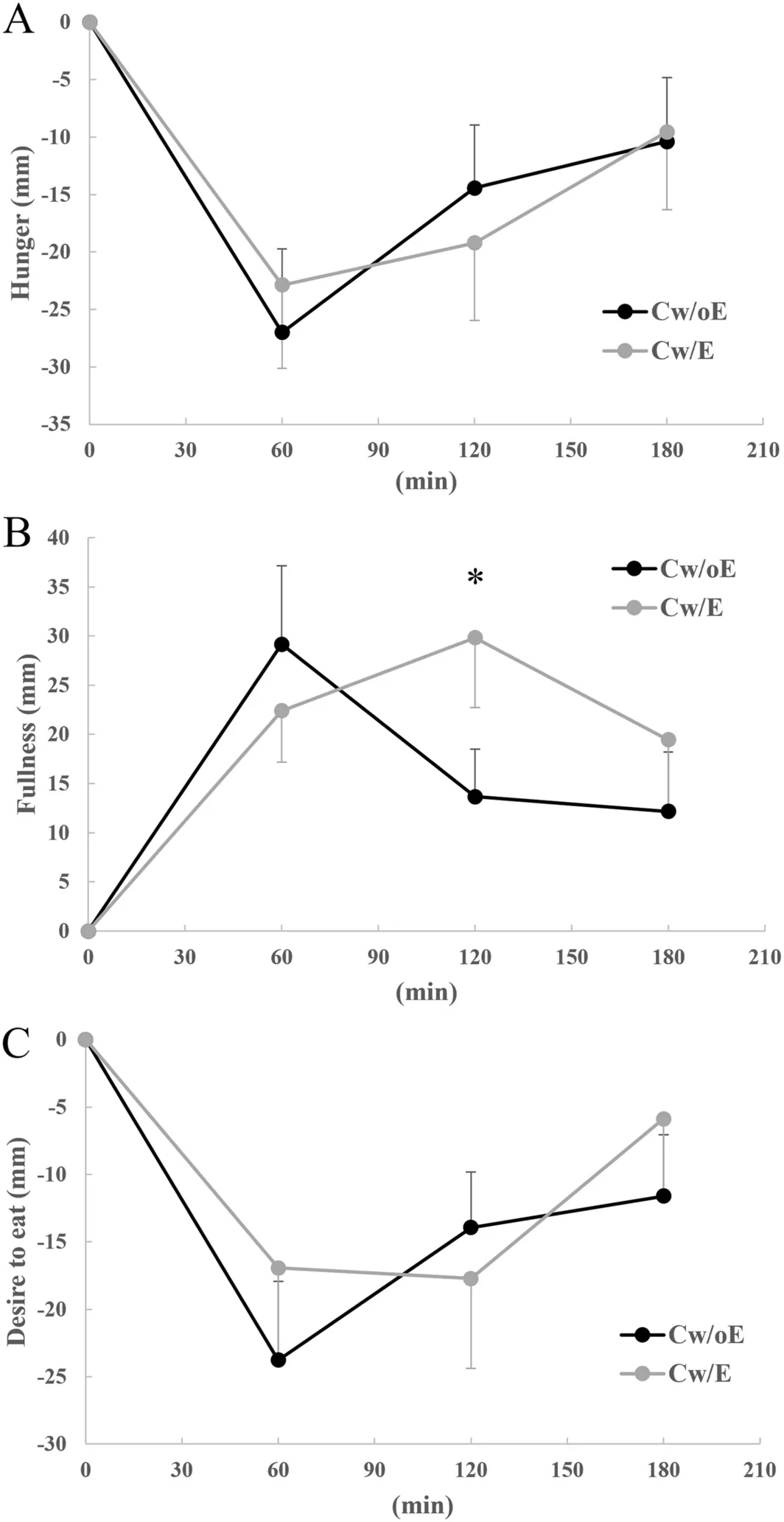
Changes in the appetite scores of participants following the consumption of chocolate processed with or without extrusion (Cw/E or Cw/oE, respectively). (A) Hunger; (B) fullness; (C) desire to eat. Error bars indicate SEM. *p < 0.05; difference between two groups.

### 3.2 In Vitro Evaluation of the Effects of Salivary and Gastric Fluids on Chocolate Composition

The concentrations of sucrose equivalent in the simulated dissolving solution in the stomach at 5, 15, and 30 min of incubation were 33.4 ± 0.9 mM, 46.7 ± 1.1 mM, and 55.0 ± 4.7 mM, respectively, in the Cw/oE group and 28.8 ± 0.9 mM, 39.0 ± 2.2 mM, and 51.9 ± 0.4 mM, respectively, in the Cw/E group. Similarly, lactose concentrations were 13.6 ± 0.9 mM, 18.1 ± 0.5 mM, and 22.6 ± 2.1 mM, respectively, in the Cw/oE group and 12.1 ± 0.3 mM, 15.8 ± 0.9 mM, and 21.1 ± 0.6 mM, respectively, in the Cw/E group. Figure 5 shows the concentration profiles of sucrose equivalent and lactose during incubation in simulated dissolving solution in the stomach, both of which increased over time but remained lower values for Cw/E, which may indicate that dissolution was inhibited as hypothesized. The amount of sucrose equivalent in Cw/E was significantly lower than that in Cw/oE at 5 and 15 min (*p* = 0.019 and 0.049, respectively), and the amount of lactose in Cw/E was lower than that in Cw/oE at 15 min, although this difference did not meet the level of significance.

**FIGURE 5:**
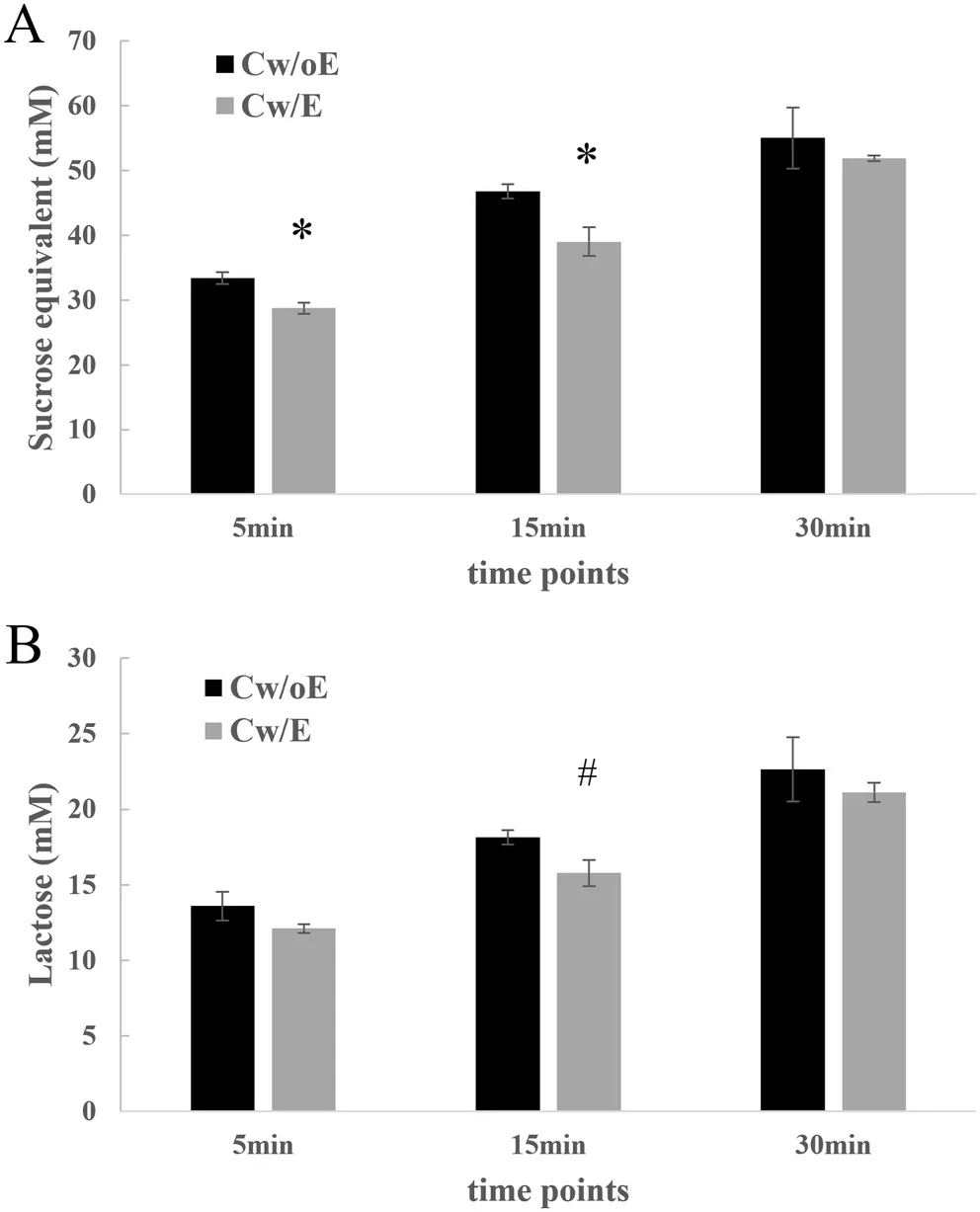
Concentration profiles of (A) sucrose equivalent and (B) lactose during incubation in simulated dissolving solution in the stomach using chocolate with or without extrusion processing (Cw/E or Cw/oE, respectively). Error bars indicate SEM. *p < 0.05; #*p* < 0.1; difference between the two groups.

## 4 Discussion

This clinical trial in healthy men revealed that different chocolate processing methods led to significant differences in blood insulin and triglyceride levels, despite the nutritional composition of the chocolate being unaffected. This suggests that extrusion processing during chocolate preparation affects the digestion and absorption of carbohydrates and lipids, supporting our hypothesis that extrusion processing is associated with the postprandial metabolic response of sugars. Although various stages of digestion and absorption, such as digestion rate, gastric emptying rate, and absorption rate, may be affected, the in vitro test results suggest that changes in the dissolution rate influence the digestion rate. Figure 5 shows that Cw/E generally exhibited lower sucrose and lactose dissolution than Cw/oE. For sucrose, the difference between the mean values of Cw/E and Cw/oE widened until 15 min, whereas the significant difference disappeared at 30 min. This difference in the dissolution speed may affect digestion in humans. Englyst et al. (2003) investigated the relationship between GI and in vitro indicators of glucose release rate from food. Rapidly available glucose is positively correlated with GI values, whereas gradually available glucose is negatively correlated with GI. In the study of drugs, the dissolution rate is considered to improve bioavailability, and studies to increase the dissolution rate are actively conducted (Kakran et al. 2012; Sandri et al. 2018; van der Merwe et al. 2020). In this study, as in the examples above, the slower dissolution of sucrose and lactose in Cw/E compared with that in Cw/oE may have decelerated the rise in blood glucose levels.

The effects of macronutrient processing on digestion and absorption have been studied (He et al. 2013; Ito et al. 2016; Miehle et al. 2024; Papakonstantinou et al. 2022). Carbohydrate bioavailability is determined by the chemical identity and physical form of food (Englyst and Englyst 2005; Golding 2019; Priyadarshini et al. 2022). The test foods used in this study are considered chemically identical in terms of macronutrients (Table 3). Regarding the difference in physical form, Ritudomphol and Luangsakul (2019) reported that adjusting the cooking temperature and water ratio created more voids and degraded the starch quickly, making it vulnerable to digestive enzymes. In this study as well, it is possible that the rearrangement of spaces such as voids of components changed depending on the conditions of extrusion processing, which affected the solubility as observed in the in vitro study, and caused the difference in blood glucose levels. In the processing of muffin dough, Miehle et al. (2024) found that in vitro glucose release was lower in the extrusion treatment under certain conditions than in the other treatments, which was attributed to the formation of a strong network between the components and an increase in the viscosity of the digestive fluid due to binding of water to the network. Moreover, in this study, it is considered that the extrusion process may suppress the release of sugar by forming a network containing sugar or increasing viscosity. Gidley and Yakubov (2019) reported that the barrier properties in food increase while food structuring, so it is possible that the release of sugar was suppressed by the barrier of tissue formed during extrusion processing in this study.

The blood parameter changes observed in this study resulted in sustained satiety. This finding suggests that Cw/E may contribute to health maintenance in terms of sustaining satiety, although its long‐term effects are unknown. Consistent with our findings, delayed sugar absorption increases satiety and reduces hunger (Ancu et al. 2025; Lavin and Read 1995). Moreover, the test substance did not include any special ingredients. The Cw/oE group demonstrated higher insulin levels than the Cw/E group, and insulin contributes to satiety through leptin secretion. However, the mechanism through which suppressed blood parameters maintain satiety has not been elucidated.

Our findings indicate that food processing can control glucose and lipid metabolism. Although the change in metabolic levels is not significant, its cumulative effect on daily consumers is relevant. Since blood glucose suppression correlates with lower nutrient absorption rates and potential weight management (Dawson et al. 2025), extrusion allows consumers to enjoy chocolate with the same taste and quantity while mitigating the increase in blood sugar. This strategy has broad implications for developing palatable, healthier foods that enhance overall well‐being. Collectively, our findings have implications for developing foods that taste good and contribute to both physical and mental health, thus promoting food well‐being.

As this study followed a crossover design, it was not necessary to consider individual differences in metabolism. However, the study had some limitations. All study participants were men and mostly middle‐aged; thus, age‐ and sex‐based differences need to be considered. Moreover, GLP‐1 levels were not evaluated, although in vitro tests were conducted.

## 5 Conclusion

Extrusion processing of chocolate significantly reduced postprandial blood glucose, insulin, and triglyceride concentrations compared to processing‐free chocolate alongside sustained feelings of fullness, relative to identical processing‐free control. Simulated dissolution analysis suggested that changes in dissolution rate influence the digestion rate in humans. In summary, we established a correlation between food processing and glucose and lipid metabolism, which has implications for developing palatable and healthy foods to promote overall food well‐being.

## Author Contributions

**Yasuyoshi Kinta:** conceptualization, methodology, project administration, resources, supervision, visualization, writing – original draft, writing – review and editing. **Hiroko Maruki‐Uchida:** data curation, formal analysis, methodology, project administration, visualization, writing – original draft, writing – review and editing. **Masahiro Umehara:** data curation, formal analysis, investigation, methodology, resources, project administration, visualization, writing – original draft, writing – review and editing. **Ryoichi Ito:** data curation, formal analysis, investigation, methodology, resources, visualization. **Ryotaro Shiga:** data curation, formal analysis, investigation, methodology, resources, visualization. **Yuki Yasumoto:** methodology, resources. **Sadao Mori:** methodology, supervision. **Takashi Koikeda:** investigation, supervision, writing – review and editing.

## Ethics Statement

The study was approved by the ethics committee of the Shiba Palace Clinic (157022–38,759) and conducted in accordance with the ethical principles of the Declaration of Helsinki (revised in 2013) and the Ethical Guidelines for Life Sciences and Medical Research Involving Human Subjects (Ministry of Education, Culture, Sports, Science, and Technology; Ministry of Health, Labor, and Welfare; and Ministry of Economy, Trade, and Industry, Japan) in January 2025.

## Consent

Written informed consent was obtained from all participants after a thorough explanation of the study purpose, content, and procedures.

## Conflicts of Interest

Yasuyoshi Kinta, Hiroko Maruki‐Uchida, Masahiro Umehara, Ryoichi Ito, Ryotaro Shiga, Yuki Yasumoto, and Sadao Mori are employees of Morinaga & Co. Ltd. The research expenses and test samples for this study were provided by Morinaga & Co. Ltd. Co‐author Takashi Koikeda is a medical doctor at the Shiba Palace Clinic who provided contractual services to conduct the clinical trial.

## Data Availability

The data presented in this study are available on request from the corresponding author.
